# Recent Progress on Functionalized Graphene Quantum Dots and Their Nanocomposites for Enhanced Gas Sensing Applications

**DOI:** 10.3390/nano14010011

**Published:** 2023-12-19

**Authors:** Thivyah Balakrishnan, Suresh Sagadevan, Minh-Vien Le, Tetsuo Soga, Won-Chun Oh

**Affiliations:** 1 Department of Chemical and Process Engineering, Faculty of Engineering & Built Environment, Universiti Kebangsaan Malaysia, Bangi 43600, Malaysia; 2 Nanotechnology & Catalysis Research Centre, University of Malaya, Kuala Lumpur 50603, Malaysia; 3 Faculty of Chemical Engineering, Ho Chi Minh City University of Technology (HCMUT), Ho Chi Minh City 700000, Vietnam; 4 Faculty of Chemical Engineering, Vietnam National University Ho Chi Minh City, Ho Chi Minh City 700000, Vietnam; 5 Department of Electrical and Mechanical Engineering, Nagoya Institute of Technology, Nagoya 466-8555, Japan; 6 Department of Advanced Materials Science and Engineering, Hanseo University, Seosan 356-706, Republic of Korea

**Keywords:** functionalized GQDs, GQDs based nanocomposite, gas sensing mechanism, improved sensing

## Abstract

Gas-sensing technology has witnessed significant advancements that have been driven by the emergence of graphene quantum dots (GQDs) and their tailored nanocomposites. This comprehensive review surveys the recent progress made in the construction methods and applications of functionalized GQDs and GQD-based nanocomposites for gas sensing. The gas-sensing mechanisms, based on the Fermi-level control and charge carrier depletion layer theory, are briefly explained through the formation of heterojunctions and the adsorption/desorption principle. Furthermore, this review explores the enhancements achieved through the incorporation of GQDs into nanocomposites with diverse matrices, including polymers, metal oxides, and 2D materials. We also provide an overview of the key progress in various hazardous gas sensing applications using functionalized GQDs and GQD-based nanocomposites, focusing on key detection parameters such as sensitivity, selectivity, stability, response and recovery time, repeatability, and limit of detection (LOD). According to the most recent data, the normally reported values for the LOD of various toxic gases using GQD-based sensors are in the range of 1–10 ppm. Remarkably, some GQD-based sensors exhibit extremely low detection limits, such as N-GQDs/SnO_2_ (0.01 ppb for formaldehyde) and GQD@SnO_2_ (0.10 ppb for NO_2_). This review provides an up-to-date perspective on the evolving landscape of functionalized GQDs and their nanocomposites as pivotal components in the development of advanced gas sensors.

## 1. Introduction

In recent years, as urban-industrial developments have accelerated, air pollution has become increasingly severe, primarily due to emissions from industrial sources including power plants, refineries, and other chemical factories [1,2,3,4]. The uncontrolled release or leakage of various hazardous gases such as volatile organic compounds (VOCs), nitrogen dioxide (NO_2_), ammonia (NH_3_), and hydrogen sulfide (H_2_S) can result in unhealthy environments and the loss of human life [5,6,7,8]. Therefore, accurate and reliable detection of toxic gases in various settings including industrial facilities, urban environments, and healthcare facilities, is necessary for ensuring public safety and maintaining good air quality. After the development of the first commercial gas sensor in 1962 [9], various types of gas sensing devices (electrochemical, infrared, catalytic, resistive, optical, photoionization, etc.) have been developed for detecting gases using different sensing materials [10,11,12,13,14,15]. Until now, a variety of materials such as metal/metal oxides, conducting polymers, metal organic frameworks (MOFs), and nanocarbon materials have been explored to satisfy the demand for gas sensors that feature high sensitivity and selectivity [6,16,17]. Among these, graphene, which was first discovered by Novoselov and colleagues in 2004 [18], has gained significant popularity as a two-dimensional (2D) material due to its unique characteristics. These properties include its thin, single atomic layer of sp^2^ hybridized carbon, excellent thermal conductivity, high electron mobility, semi-metallic nature, and exceptional mechanical tensile strength [19,20,21]. Notably, graphene is considered as a viable gas-sensing material due to its electronic characteristics, which are significantly influenced by gas molecule adsorption [22]. However, the absence of an intrinsic electronic bandgap or of functional groups in pristine graphene limits its use in gas-sensor applications [23]. Graphene’s lack of a bandgap refers to the energy difference between the valence band and the conduction band being nearly zero or very small [24]. For gas-sensing applications, having a bandgap is beneficial because it allows the material to selectively interact with specific molecules, leading to changes in its electronic properties in the presence of those molecules. In the absence of a bandgap, pristine graphene may not exhibit significant changes in its electronic structure when exposed to certain gases, making it less effective for gas-sensing applications. Furthermore, in the absence of functional groups, pristine graphene may not have enough sufficient interactions with gas molecules to produce a detectable response [25]. When graphene, originally in a 2D form, is converted into zero-dimensional quantum dots (QDs), it undergoes a reduction in its lateral dimensions to the nanometer range (typically between 2 and 10 nm), and its thickness is reduced to approximately 1–2 nm, resulting in the formation of graphene quantum dots (GQDs) [18,26].

GQDs offer a combination of advantages from both their graphene and QD components, including the 2D and quantum confinement effects [27,28,29]. This integration results in several outstanding features such as remarkable optical transparency, large surface area, unique photoluminescence, biocompatibility, minimal toxicity, high chemical stability, and a customizable energy band gap that can suit specific requirements [30,31,32]. In comparison to 2D graphene, the band gap of GQDs is readily tunable over an extensive range due to their unique quantum limitations and edge effects [33]. Additionally, GQDs, with their ultra-small particle size, provide an abundance of oxygen-containing functional groups, vacancies, and defects. These characteristics make GQDs the preferred choice for gas-sensing applications, especially when combined with other gas-sensing materials to form p–n or p–p heterojunctions, thereby enhancing the detection of toxic gases. Incorporating GQDs with other materials such as metal oxides [34,35], polymers [36], and MOFs [37,38] to create GQD-based nanocomposites with synergistic effects is a powerful strategy for enhancing sensor performances and can lead to improved sensitivity, selectivity, stability, and tunability. Furthermore, compared to pristine graphene, the presence of hydroxylated functional groups in GQDs enhances their hydrophilicity and offers extensive possibilities for surface functionalization [39]. Surface functionalization with organic molecules and doping with elements such as sulfur (S), nitrogen (N), phosphorus (P), boron (B), silicon (Si), and magnesium (Mg) significantly enhances the optical characteristics, electronic properties, and chemical reactivity of GQDs, allowing for the fine-tuning of their inherent properties for specific gas-sensing applications [40,41,42]. These exceptional characteristics have accelerated a rapid progress in the development of functionalized GQDs and GQD-based nanocomposites, especially within a wide range of sensing applications.

Numerous researchers have recently reviewed and published findings on the chemical and physical properties of GQDs and their synthesis methods [39,43,44]. Some of the reviews have focused on the applications of GQDs and their nanocomposites in drug delivery [45,46], bioimaging [46,47], wastewater treatment [48,49], food safety [50], energy storage [51,52], and catalysis [53]. Although there are existing reviews on gas detection, there is still a scarcity of reviews that specifically address the application of GQDs and their nanocomposites for toxic gas sensing. Therefore, to promote the practical use of GQD-based gas sensors, we hereby present this review which covers an overview of recent advancements in functionalized GQDs and GQD-based nanocomposites as toxic gas sensors. First, we briefly explain strategies for the development of functionalized GQDs and GQD-based nanocomposites. Subsequently, we provide an overview of the key progress in various hazardous-gas-sensing applications using functionalized GQDs and GQD-based nanocomposites, focusing on sensing mechanisms and key detection parameters, such as sensitivity, selectivity, stability, response and recovery time, repeatability, and limit of detection (LOD). Finally, we discuss the challenges and prospects of the GQD-based gas sensors for further development and practical applications.

## 2. General Construction Methods of GQD-Based Gas Sensors

GQDs can be synthesized using either a top-down or bottom-up approach, depending on the type of precursor used [54,55]. Comprehensive reviews addressing these topics are readily available in the literature [39,46,52,56]. In this review, we briefly outline the construction strategies for functionalized GQDs and GQD-based nanocomposites tailored for gas-sensing applications. Researchers have actively explored strategies for tuning the chemical properties of GQDs through elemental doping and the modification of the functional groups on their edges and surfaces, such as hydroxyl, carboxyl, or amino groups, with the aim of enhancing the GQDs gas-sensing capabilities [57,58]. In addition, the functionalized GQDs are also incorporated with certain materials to meet the requirements of specific gas-sensing applications. Table 1 summarizes the different types of GQD-based sensors, including their preparation methods and target gases. It was observed that GQDs can be composited with other functional materials through methods such as stirring, coating, hydrothermal reactions, π–π stacking, chemical oxidative polymerization, and ultrasonic impregnation.

**Figure 1:**
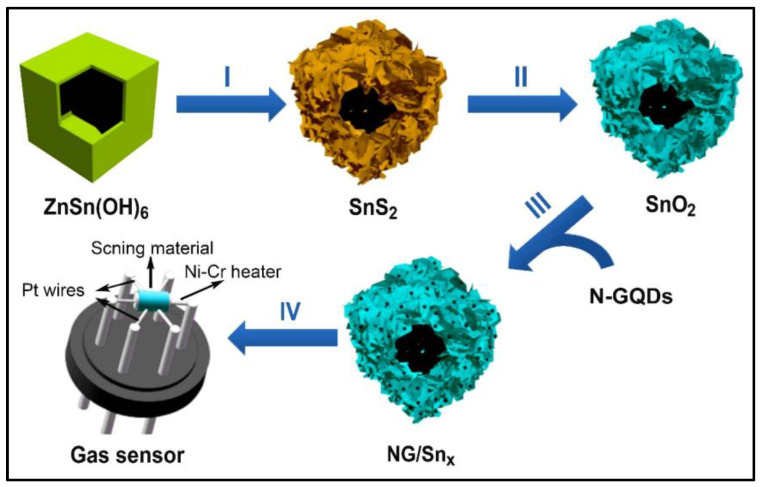
Synthetic schematic diagram of N-GQDs@SnO_2_ nanocomposites [66]. I–IV represent the steps of the process order.

Lv et al. successfully prepared a N–GQDs@SnO_2_ composite by vigorously stirring pre-synthesized N–GQDs and SnO_2_ to detect NO_2_ gas at a working temperature of 130 °C [66]. The sensor demonstrated outstanding detection ability (Rg/Ra = 25.3) for low-concentration NO_2_ (100 ppb). Figure 1 shows the synthetic schematic diagram of N-GQDs@SnO_2_. Although the stirring method has advantages in terms of simplicity and cost-effectiveness, it may not provide precise control over the structure and distribution of the GQDs within the composite. Rahul et al. also developed an N–GQDs@SnO_2_ composite for the detection of NO2 gas, but they employed a different synthesis approach, namely, ultrasonic impregnation [69]. This N–GQDs@SnO_2_ composite exhibits an enhanced response (Rg/Ra = 292) with a short response (181 s) and recovery time (81 s) toward 100 ppb NO_2_ gas at 150 °C. From these reports, it can be concluded that the gas-sensing performance of the sensing materials can be significantly influenced by the choice of synthesis method.

Ebrahimi et al. synthesized ZnCo_2_O_4_/GQD’s coral-like nanostructures using a simple hydrothermal method that showed good selectivity for triethylamine [60]. The hydrothermal method offers advantages such as high particle dispersion, ease of synthesis, and cost-effectiveness [47]. However, it often requires specialized equipment, including high-pressure reactors and controlled temperature environments, and can be time-consuming.

**Figure 2:**
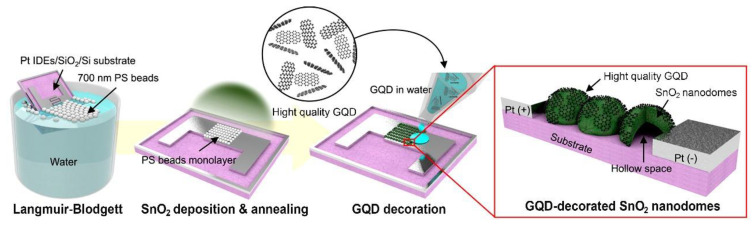
Fabrication process for a GQD@SnO_2_ nanodome based gas sensor [61].

Chen et al. utilized an ultrasonic impregnation technique to synthesize well-dispersed N-GQDs@SnO_2_ composites for high-efficiency HCHO detection [68]. Ultrasonic impregnation is effective in achieving a uniform dispersion of GQDs within the composite matrix; however, it may not offer the same level of control over the GQDs’ sizes and structures as is offered by other synthesis methods, such as hydrothermal synthesis [70]. Additionally, Jiang et al. constructed the CoPc–GQD composite as a DMMP detection sensor based on the π–π stacking approach [63]. The π-π stacking technique is relatively simple, relying on non-covalent interactions between GQDs and other materials, and it does not involve complex chemical reactions. However, this technique can limit the stability of the composite, especially when exposed to harsh conditions such as high temperatures [71]. Lee et al. reported NO_2_-responsive GQD@SnO_2_ nanodomes by drop-casting a GQD solution onto SnO_2_ nanodomes and subsequently drying it at room temperature for 24 h as shown in Figure 2 [61]. Drop-casting is a straightforward and cost-effective technique, but it may cause aggregation or uneven distribution of GQDs, which can affect the properties of the composite.

## 3. Gas-Sensing Mechanisms of GQD-Based Sensors

Gas-sensing mechanisms in GQD-based sensors involve several key processes that enable the detection of and response to specific gas molecules [72]. These mechanisms can vary depending on the type of gas and the specific configuration of the GQD-based sensor. Some common gas-sensing mechanisms associated with GQD-based sensors are based on the Fermi-level control and charge carrier depletion layer theory, which is explained by the formation of heterojunctions [61,73] and the adsorption/desorption principle [62,67].

### 3.1. Heterojunction

In general, GQDs are frequently combined with other materials such as metal oxide semiconductors, organic polymers, and transition metal dichalcogenides to significantly enhance their sensing capabilities [34,35]. This enhancement arises from the distinct energy band structures of GQDs and hybrid materials, which prompt the transfer of electrons or holes at the interface between these components until their Fermi levels reach equilibrium at the same energy level [74]. Consequently, heterojunctions are formed at the interface between the GQDs and the hybrid materials, which play a crucial role in adjusting the thickness of the depletion/accumulation layer and the height of the potential barrier [23,75,76]. This, in turn, alters the internal distribution of electrons among the different components and profoundly impacts the sensing performance of the materials. When investigating the mechanisms of GQD nanocomposites, it is imperative to primarily consider the influence of the heterojunctions. In this section, we elucidate the mechanism of GQD-based nanocomposites through two subsections: anisotype heterojunctions (p–n) and isotype heterojunctions (n–n, p–p).

In p–n heterojunctions, in which the n-type material has a higher Fermi energy level than the p-type material, electrons migrate from the n-type material to the p-type material, while the holes move in the opposite direction until their Fermi energy levels equalize [77]. This process leads to the formation of a depletion layer at the interface and the bending of the energy bands, resulting in a potential barrier that narrows the electron transport channel [23]. In the case of isotype heterojunctions (n–n, p–p), the phenomenon of band bending also occurs as a result of differences in Fermi energy levels [78]. In n–n heterojunctions, electrons transfer from the region with higher Fermi energy levels to the region with lower Fermi energy levels, leading to the formation of an electron depletion layer on the higher Fermi energy side and an electron accumulation layer on the lower Fermi energy side [76]. Similarly, in p–p heterojunctions, holes transfer from the region with lower Fermi energy levels to the region with higher Fermi energy levels, resulting in the formation of a hole accumulation layer on the higher Fermi energy side and a hole depletion layer on the lower Fermi energy side [77]. Notably, the n–n and p–p heterojunctions contribute to the formation of an electron depletion layer and a hole depletion layer, respectively, resulting in an enhanced sensing response and excellent response/recovery performance [79]. 

### 3.2. Chemisorption

Chemical adsorption and desorption represent prominent gas-sensing mechanisms that exert a significant influence on most GQD-based gas sensing devices. When a gas directly interfaces with a sensor, a chemical reaction occurs, leading to alterations in electrical signals [80]. This change can result from the presence of the target gas or from ambient oxygen molecules. Oxygen adsorption is among the most prevalent gas-sensing mechanisms and has a profound impact on most GQD-based gas sensing devices [3,14]. When the sensor is exposed to air, oxygen molecules begin to adsorb onto the material’s surface, initiating oxidization or reducing reactions between the atmospheric oxygen and the sensing surface. These reactions give rise to substantial changes in certain electrical properties or the resistance of the sensing material [81,82]. Depending on the operating temperature, various oxygen ions (O_2_^−^, O^−^, and O^2−^) are generated after capturing electrons from the sensing materials. Consequently, changes in surface electron density leads to variations in the conductivity or resistance of the sensing material.

Upon exposure to reducing target gases on n-type GQD-based sensors, the electrons captured by oxygen species are released back into the sensing material, resulting in a decrease in resistance [83,84]. This reduction in resistance is further confirmed by a decreased barrier height at the interface. Conversely, when oxidizing gases are present, the electron density decreases, causing an increase in resistance. In contrast, when reducing gases are adsorbed onto the surface of a p-type GQD-based sensor, the hole accumulation layer diminishes due to electron–hole recombination processes [81,85]. Consequently, the surface resistance of the p-type sensor increases. However, in the presence of oxidizing gases, the hole carrier concentration significantly rises due to the trapping of electrons by the oxidizing gases, which leads to a decrease in resistance.

## 4. Role of GQDs for Enhanced Gas Sensing

GQDs play pivotal roles in achieving exceptional gas-sensing performances in GQD-based sensors. In this section, we delve into an analysis of the enhanced gas-sensing performance by elucidating the various roles that GQDs play in gas detection. Specifically, GQDs serve four vital functions as follows: (i) Strong interaction with the target gas [34,64]. GQDs exhibit a remarkable capacity to establish robust interactions with the target gas molecules. (ii) Formation of heterojunction [86,87]. This heterojunction formation is of paramount importance, as it facilitates improved charge transfer and electron mobility, thereby enhancing the sensor’s overall performance. (iii) Increased surface area [60,65]. This larger surface area offers more active sites for the adsorption and diffusion of gas molecules. (iv) Protective layer [62]. GQDs’ shield-sensitive sensor components from environmental factors have the ability to contribute to the sensor’s reliability and durability. The roles of GQDs in improving the sensing performance of reported GQDs based gas sensors are summarized in Table 2.

### 4.1. Strong Interaction with Analyte

**Figure 3:**
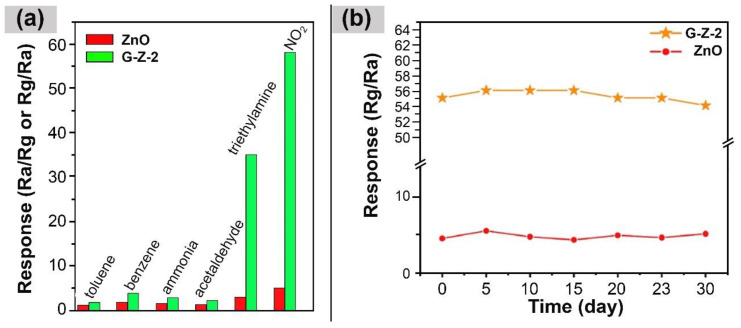
(**a**) Responses of ZnO and N-GQDs@ZnO (G-Z-2) to 5 ppm of different gases at 100 °C; (**b**) Long-time stability of sensors to 5 ppm NO_2_ [67].

A strong interaction with the analyte plays a crucial role in enhancing gas-sensing performance, ultimately enabling the development of highly reliable sensors capable of detecting toxic gases even at extremely low concentrations [96,97]. By utilizing surface functionalization and tailoring the electronic properties, it is possible to optimize GQDs for specific gas-sensing applications, promoting strong interactions with target gas molecules, and improving sensitivity [5]. Zhang et al. investigated the NO_2_ gas-sensing properties of a hydrothermally synthesized N–GQDs@ZnO nanocomposite [67]. The nanocomposite exhibited a remarkable 11.6-fold enhancement in sensitivity to 5 ppm NO_2_ (Figure 3), achieving a detection limit of 0.1 ppm while also reducing the working temperature from 160 °C to 100 °C. The higher sensitivity to NO_2_ is attributed to the doping of electronegative N atoms, a process in which the electron-attracting NO_2_ molecules preferentially bind to the N atoms due to the basicity of the N-containing groups. This enhanced adsorption of NO_2_ on the surface of N–GQDs@ZnO significantly increases the charge transfer between the NO_2_ molecules and the N-GQDs’ surface.

In addition to this, the introduction of GQDs to various functional groups increases the oxygen vacancy content in the material, thereby significantly boosting the concentration of free charges and promoting electron transfer on the material’s surface [98]. Chen et al. demonstrated this phenomenon by developing a N–GQDs/SnO_2_ nanocomposite for highly sensitive HCHO sensing, achieving a detection limit of 0.01 ppm [68]. When the N–GQDs were attached to SnO_2_ nanosheets, electrons were transferred from the N–GQDs (which have a low work function) to the SnO_2_ (which have a high work function), resulting in the creation of a Schottky barrier between them. Consequently, this led to an increase in the adsorption of oxygen molecules and the thickening of the electron depletion layer, potentially causing an increase in resistance as compared to the pristine SnO_2_. The authors claimed that the improved sensing properties of the N–GQDs were attributable to the presence of numerous functional groups, enhanced oxygen adsorption, and the electronic regulation of the SnO_2_ nanosheets [68].

### 4.2. Formation of Heterojunction

**Figure 4:**
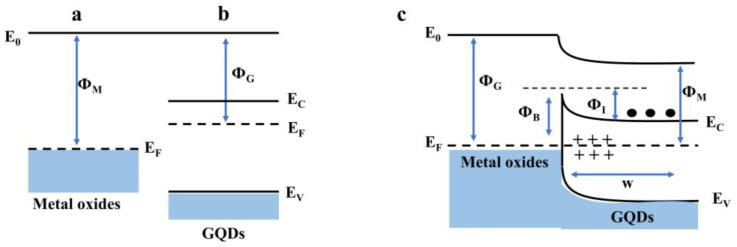
Schematic illustration of the heterojunctions formed by GQD-metal oxides (G/M). (**a**) Metal oxides work function, Φ_M_, and Fermi energy, E_F;_ (**b**) GQDs’ work function, Φ_G_; (**c**) Idealized equilibrium band diagram for the G/M junction. Φ_i_ is the energy barrier to the flow of electrons (black dots) from the GQDs to the metal oxides, while Φ_B_ is the Schottky barrier height for the electron flow in the opposite direction. w is the extension of the depletion layer and corresponds to the bent part of the energy bands Reproduced from [99].

GQDs have the ability to create a heterojunction interface which enhances gas-sensing performance by having different Fermi levels between two components. To achieve a strong electrical field at this interface, the Fermi levels on either side can be aligned, enabling the electrons to transfer from the component with a higher Fermi level to the one with a lower Fermi level. This process facilitates charge transfer and expands the area of charge depletion, which further improves the gas-sensing properties [74,99]. Figure 4 shows a schematic illustration of the energy and structures of the GQD-metal oxides junction and the electron transfer in the nanocomposite.

**Figure 5:**
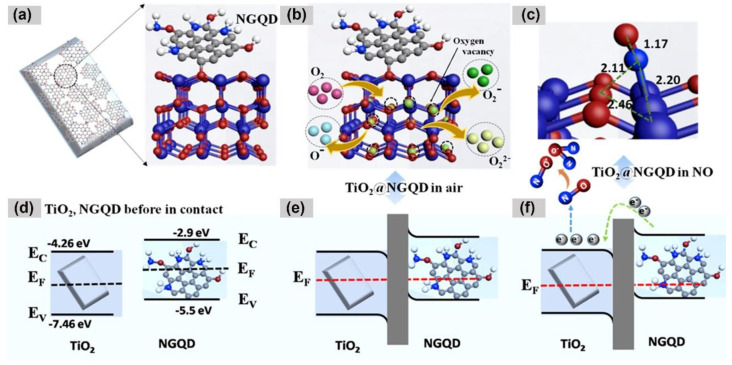
Schematic illustration of (**a**) TiO_2_@NGQDs’ hybrid formation; (**b**) O_2_ adsorption and conversion to the oxygen ion species on TiO_2_; (**c**) the most stable configuration of NO gas adsorbed on to the TiO_2_ surface; schematic illustration of the energy and structures of the TiO_2_@NGQDs p−n junction and the electron transfer in the nanocomposite; (**d**) TiO_2_ and NGQDs before contact; (**e**) TiO_2_@NGQDs nanocomposite in air; and (**f**) exposure to NO. EC, EV, and EF are the conduction band, valence band, and Fermi energy, respectively [86].

For example, Murali et al. synthesized a NO gas sensor by decorating TiO_2_ nanoplates with NGQDs through precursor graphitization by using a hydrothermal approach [86]. At ambient temperature, the TiO_2_@NGQDs hybrid demonstrated a 12.0% response to 100 ppm NO, marking a 4.8-fold increase as compared to the response of the pure TiO_2_ nanoplates. The improved gas-sensing capabilities of TiO_2_@NGQDs, in contrast to TiO_2,_ can be ascribed to the formation of heterojunctions between TiO_2_ and NGQDs as illustrated in Figure 5. The free electrons are transferred from n-type TiO_2_ with a high work function of 4.26 eV to p-type NGQDs with low work function of 2.9 eV, while the holes move in the opposite direction, until equilibrium is achieved. Consequently, an internal electric field is established at the interface of the TiO_2_ and NGQDs heterojunction, leading to band bending in the depletion layers and increased conductivity. Apart from the p–n heterojunction, Yumin et al. demonstrated benzene gas detection based on a boron-doped graphene quantum dot (BGQDs)/Ag–LaFeO_3_ (B/APPH) p–p heterojunction [87]. In air, the absorbed oxygen molecules can capture electrons from the surface of Ag-LaFeO_3_, resulting in low resistance. Upon exposure to benzene gas, the released electrons recombined with electron holes from the hole accumulation layer and the resistance was increased significantly. Therefore, the presence of the p–p heterojunction between the BGQDs and the Ag-LaFeO_3_ enhanced carrier transport capabilities lowered the operational temperature to 65 °C, maintaining a strong sensing response (17.5) and good selectivity.

### 4.3. Higher Surface Area

Materials with large surface areas are highly preferred for the development of gas sensors that are compact, cost-effective, energy-efficient, and exceptionally sensitive. GQD-based sensors featuring enlarged surface areas demonstrate superior capabilities in detecting trace gas concentrations, which makes them particularly suitable for applications demanding precision and reliability [100]. In a study by Ebrahimi et al., a ZnCo_2_O_4_/GQD coral-like nanostructure exhibited a remarkable response (6.97–100 ppm) toward triethylamine with a low detection limit of 0.43 ppm [60]. The scanning electron microscope (SEM) images of the ZnCo_2_O_4_ and ZnCo_2_O_4_/GQDs confirmed that both nanocrystals have coral-like shapes with porous surfaces. The ZnCo_2_O_4_/GQDs exhibited a smaller grain size compared with the ZnCo_2_O_4_. When compared to the coral-like ZnCo_2_O_4_ nanostructure, the composite structure of the coral-like ZnCo_2_O_4_/GQDs showcased significantly enhanced gas-sensing properties. These improvements were attributed to their larger pore volume (0.46 cm^3^ g^−1^) and diameter (32.1 nm) relative to the nanocrystalline ZnCo_2_O_4_.

**Figure 6:**
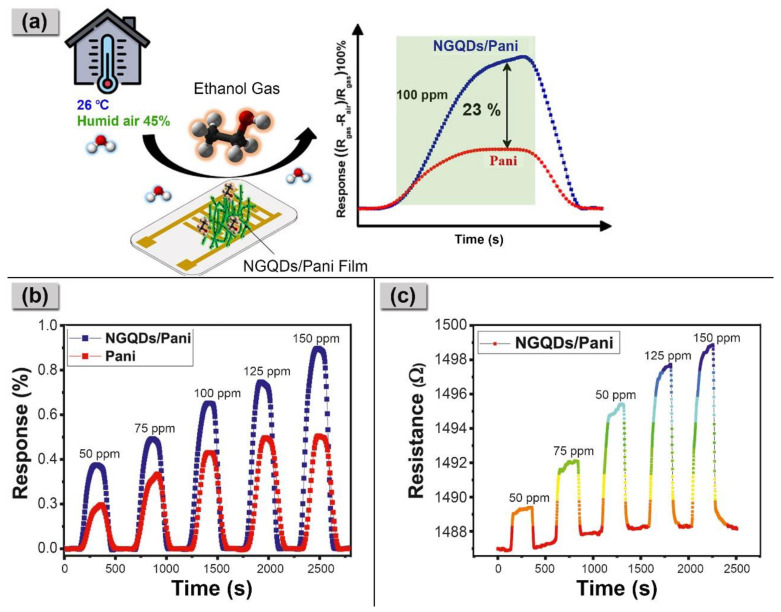
(**a**) Improved gas response of the NGQDs/PANI composite toward 100 ppm ethanol; (**b**) Gas response of PANI and NGQDs/PANI film sensors toward 50–150 ppm of ethanol at 26 °C in 45% RH; (**c**) real-time resistance change as a function of time for the NGQDs/PANI film sensor toward ethanol gas [65].

Additionally, Masemola et al. utilized an in situ chemical polymerization method to synthesize NGQDs/PANI composite sensors [65]. These sensors exhibited a substantially higher response, up to 23%, as compared to that of pure PANI for 100 ppm ethanol, as demonstrated in Figure 6a. Moreover, Figure 6b illustrates that both sensors displayed linear responses with increasing concentrations of ethanol gas (ranging from 50 to 150 ppm). However, the NGQDs/PANI composite sensor achieved a greater response of 39%. Similar results were obtained for the real-time resistance change as a function of time when the NGQDs/PANI composite sensor was exposed to 50–150 ppm of ethanol vapors (Figure 6c). The incorporation of NGQDs in PANI led to enhanced sensitivity and resulted in the lowest response and recovery times of 85 s and 62 s, respectively, when exposed to 100 ppm of ethanol. This enhancement can be attributed to the higher surface porosity of the NGQDs/PANI composite, which provided more active sites for the ethanol gas molecules to adhere to its surface, leading to increased gas adsorption.

### 4.4. Protecting Layer

**Figure 7:**
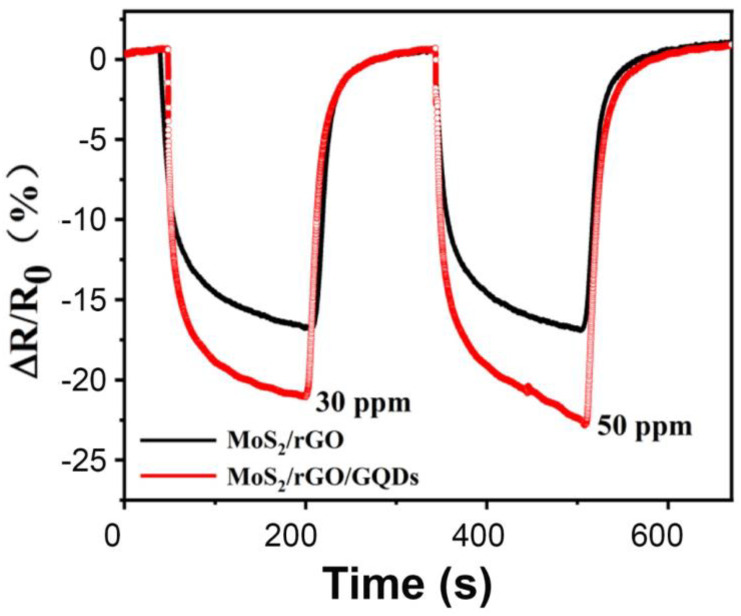
Response and recovery curves of MoS_2_/rGO and MoS_2_/rGO/GQD-based sensors exposed to 30 and 50 ppm NO_2_ [62].

Due to their chemical inertness and high stability, GQDs can serve as a protective layer to prevent the agglomeration of metal oxide nanocomposites and the oxidation of silicon nanowire-based gas sensors. Yang et al. prepared MoS_2_/rGO/GQDs ternary hybrids for the detection of NO_2_ gas [62]. The aggregation of MoS_2_ nanoflowers weakens the supporting effect of the rGO nanosheets and reduces the probability of NO_2_ gas adsorption on the heterogeneous interface between the MoS_2_ nanoflowers and the rGO nanosheets, thereby causing a decrease in gas sensitivity. To address this issue, the authors introduced GQDs to provide nucleation sites for the formation of MoS_2_/rGO nanocomposites, which improved the homogeneous distribution of the rGO and MoS_2_ nanosheets and prevented their agglomeration. Simultaneously, the GQDs also acted as active sites, providing numerous reaction sites for NO_2_ gas adsorption and leading to the improved gas-sensing performance of the hybrids. In comparison to the MoS_2_/rGO nanocomposite, the addition of GQDs enhanced sensitivity from 16.8% to 21.1% and from 16.9% to 23.2% when the sensor was exposed to 30 and 50 ppm NO_2_ gas at room temperature, respectively (Figure 7). Moreover, it maintained a consistent response of 23.2% even after three consecutive cycles, demonstrating the outstanding stability and repeatability of the MoS_2_/rGO/GQDs hybrid.

### 4.5. Enhanced Selectivity

The gas sensor’s selectivity is a crucial aspect, denoting the ability of the sensing materials to discern and detect a specific gas within a complex mixture of various gases, which are commonly referred to as interfering gases [5]. In this context, the “target gases” are those particular gases which the sensor is designed to identify and measure amidst the presence of other gases. The doping of GQDs and the formation of composites have a significant impact on the selective detection of target gases. Rahul et al. developed a N–GQDs@SnO_2_ heterostructure that exhibits excellent selectivity toward NO_2_ over other interfering gases (SO_2_, H_2_S, CO, and NH_3_) [69]. The sensor’s response to 1 ppm NO_2_ at 150 °C is significantly higher than its responses to other gases at the same concentration, indicating the robust selectivity of the N–GQDs@SnO_2_-based NO_2_ sensor. This superior selectivity is attributed to the N-doped GQDs, which possess a lower binding energy for NO_2_ as compared to pristine. The N atoms incorporated into the GQDs serve as selective active sites for NO_2_ adsorption, enabling the sensor to selectively detect NO_2_ even at low concentrations as compared to other tested gases. Yumin et al. fabricated boron-doped GQDs with benzene-imprinted Ag–LaFeO_3_ to develop a benzene sensor (BI-AL) with a high response, good selectivity, and low operating temperature [87]. The BI–AL sensor exhibits a highly selective detection of 1 ppm benzene at 125 °C despite the presence of other interfering gases including formaldehyde, ammonia, acetone, toluene, gasoline, methanol, and ethanol. The authors claim that several recognition cavities, complementary to benzene in shape, size, and chemical functionality, can selectively adsorb benzene and thereby improve the sensor’s selectivity. In their study, they mixed the benzene template (benzene) with functional monomers (FA) to form a benzene–FA complex through hydrogen bonding. The resulting complex was subsequently copolymerized with a large excess of crosslinker (Ag-LaFeO_3_ sol). Finally, after removing the template, recognition cavities complementary to benzene molecules were formed and exhibited a high recognition and binding ability for benzene. This resulted in an improvement in the selectivity of the BI–AL sensor.

### 4.6. Synergistic Effects

**Figure 8:**
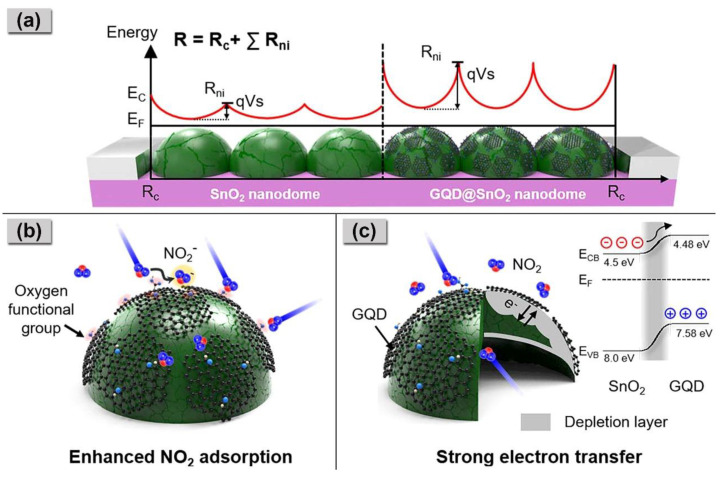
Schematic illustration of (**a**) the initial potential barrier formation for the SnO_2_ nanodomes structure; (**b**) the sensing mechanism of GQD@SnO_2_ nanodomes which shows enhanced NO_2_ adsorption due to the GQDs; (**c**) the formation of an electron depletion layer with its electronic band structure [61].

The synergistic effects of GQDs with multiple roles in gas sensing offer enhanced sensitivity, selectivity, response times, and overall performance of gas sensors as compared to the single effect. These advancements are crucial for addressing the growing need for accurate and efficient gas detection in fields ranging from environmental monitoring to industrial safety. Lee et al. discovered that GQDs could establish a robust interaction with the target gas while simultaneously forming a heterojunction to enhance electron transfer. They reported that the GQD@SnO_2_ nanodome gas sensor demonstrated an improved NO_2_ gas-sensing performance at room temperature with an extremely low detection limit of 1.1 ppb. This enhancement is attributed to the increased adsorption energy of NO_2_ gases, which is primarily influenced by the oxygen-functional groups on the GQDs, as illustrated in in Figure 8 [61].

According to density functional theory studies, the calculated adsorption energy of NO_2_ on a SnO_2_ surface is approximately −0.52 eV, whereas on hydroxyl groups of GQDs, the calculated adsorption energy reaches −0.91 eV, which implies a strong interaction between the functional groups within GQDs and NO_2_ molecules. Moreover, the formation of a p–n heterojunction between GQDs and SnO_2_ facilitates electron transfer from the n-type SnO_2_ to the p-type GQDs, expanding the electron depletion layer on the surface and consequently leading to effective resistance modulation. Similarly, Lv et al. discovered that the highly enhanced NO_2_ sensing behavior of the synthesized N-GQD@SnO_2_ is primarily attributed to the formation of heterojunctions between N–GQDs and SnO_2_ [66]_._ Additionally, the presence of doped N atoms on the surface of GQDs provides more active adsorption sites for NO_2_ due to the atoms’ strong electrophilic ability. The synergistic effect induced by the GQDs results in an improved response to 1 ppm NO_2_, which is approximately 2.2 times greater than that of pure SnO_2_ at 130 °C.

**Figure 9:**
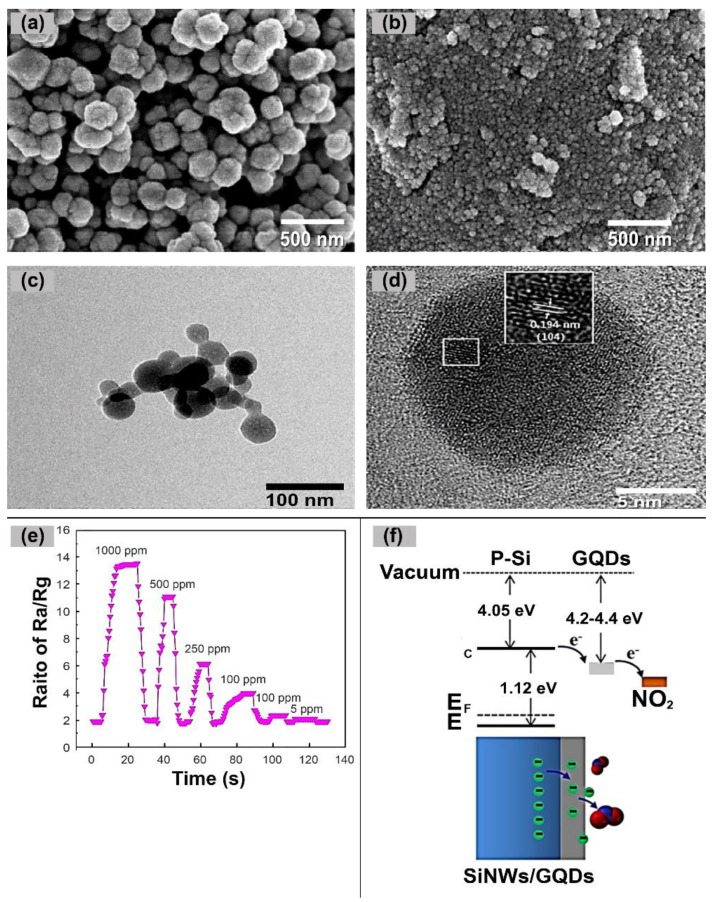
(**a**) The SEM image of pure ZnFe_2_O_4_; (**b**) the SEM image of ZnFe_2_O_4_–GQDs; (**c**) the TEM images of ZnFe_2_O_4_–GQDs; (**d**) the HRTEM images of ZnFe2O4–GQDs; (**e**) The response of the ZnFe_2_O_4_–GQDs composite to acetone (1000, 500, 250, 100, 10 and 5 ppm) at room temperature; (**f**) Energy band diagram of the GQDs/SiNW heterojunction. Figure 9a–e were adapted from [90] and Figure 9f was adapted from [89].

In another study, Chu et al. confirmed that GQDs played a dual role in significantly influencing the acetone gas-sensing responses and selectivity of ZnFe_2_O_4_–GQDs nanocomposites at room temperature [90]. The incorporation of GQDs provided a larger specific surface area for the ZnFe_2_O_4_–GQDs composites as compared to the pristine ZnFe_2_O_4_ composites, resulting in more active sites for the adsorption and diffusion of the acetone molecules and fast carrier transport. As shown in Figure 9a,b, the size of the pristine ZnFe_2_O_4_ ranged from 50 to 250 nm, which was larger than the size of the ZnFe_2_O_4_–GQDs (20–50 nm). Figure 9c indicated that the addition of GQDs in the ZnFe_2_O_4_–GQDs composite impeded the agglomeration of ZnFe_2_O_4_ crystals and contributed to the growth of smaller grains. The HRTEM image of ZnFe_2_O_4_–GQDs (Figure 9d) shows the lattice spacing of the (104) plane of graphitic carbon (about 0.194 nm), affirming the successful incorporation of GQDs with ZnFe_2_O_4_. The resulting nanocomposite exhibited responses of 13.3 and 1.2 to 1000 ppm and 5 ppm of acetone, respectively, and demonstrated quick response and recovery times (less than 12 s) as shown in Figure 9e.

In addition, GQDs can serve as a protective layer and form a heterojunction to enhance gas-sensing performance. Li et al. incorporated GQDs with silicon nanowires (SiNWs) to protect the SiNWs from oxidation and enhance the carrier interaction with analytes [89]. The silicon surface is highly susceptible to oxidation, leading to the formation of SiOx. In such cases, the oxide layer can impede the transfer of charges between the silicon and analyte, potentially disrupting the sensing functionality. By preventing oxidation and preserving the integrity of the SiNWs, GQDs contributed to the improved performance of the NO_2_ gas sensor. Furthermore, the GQD/SiNW heterojunction (Figure 9f) facilitated rapid electron transfer from the composite to the absorbed NO_2_ molecules due to their high electron-withdrawing ability and the abundant electron storage capacity in the GQDs layer. Therefore, in contrast to the bare SiNW array, the GQD/SiNW sensor demonstrated exceptional sensitivity for detecting trace amounts of NO_2_ (as low as 10 ppm) at room temperature. The authors demonstrated that GQDs not only protected the SiNW array from oxidation but also improved the electron interactions between the detector and analytes, benefiting both the response and recovery processes during detection.

## 5. Performance of GQD-Based Gas Sensors

The following subsections highlight some of the works conducted by various researchers in the detection of different types of gases. The gas-sensing performances of graphene, GQDs, and other nanoparticle (NP)-based sensors for various gases are discussed. Table 3 presents comparisons of these sensors based on pivotal electrochemical gas-sensing parameters including sensitivity, response and recovery times, operating temperature, and detection limits.

### 5.1. Detection of NO_2_ Gas

**Figure 10:**
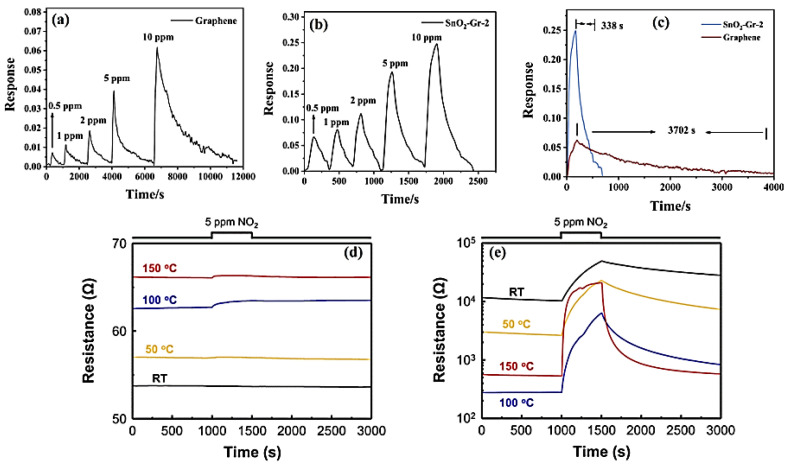
Response curves to different concentrations of the sensors’ NO_2_ based on (**a**) graphene and (**b**) SnO_2_–Gr–2; (**c**) the response and recovery times of the sensors [121] and resistance curves for 5 ppm NO_2_ as a function of operating temperature for (**d**) pristine SnO_2_ nanodomes and (**e**) a GQD@SnO_2_ nanodome-based gas sensor [61].

Zhang et al. developed a SnO_2_/graphene nanocomposite, denoted as SnO_2_–Gr–2, for the detection of NO_2_ gas [121]. The monolayer graphene was prepared using the chemical vapor deposition method and subsequently decorated with SnO_2_ through a drop-casting and vacuum annealing process. The SnO_2_–Gr–2 sensors exhibited a sensitivity nearly four times that of the pure graphene sensor, and they achieved an almost 11-fold reduction in recovery time, as depicted in Figure 10a–c, respectively. Pristine graphene is characterized by longer recovery times (3702 s) due to the strong adhesion of NO_2_ molecules to its surface. The hybridization of graphene with SnO_2_ not only promotes the rapid desorption of NO_2_, resulting in shorter recovery times (338 s), but also creates more active adsorption sites for NO_2_ at the SnO_2_–graphene heterojunction. This, in turn, leads to a rapid change in electrical conductivity, ultimately enhancing the sensing performance. In another study, Lee et al. successfully decorated SnO_2_ with GQDs (GQD@ SnO_2_) for enhanced NO_2_ gas detection over a wide operating temperature range from room temperature to 150 °C [61]. The GQD@ SnO_2_ nanodome exhibited a notable response to 5 ppm NO_2_ gas ((R_g_/R_a_) − 1 = 4.8) at room temperature, while the pristine SnO_2_ nanodomes showed no response, as demonstrated in Figure 10d. Furthermore, a 30-times higher response to NO_2_ was obtained at 150 °C as compared to the pristine SnO_2_ nanodomes. Additionally, the GQDs decoration significantly improved the recovery time, reducing it from 1247 s for the bare SnO_2_ nanodomes. The GQD decoration achieved these enhancements by increasing the potential barrier between the nanodomes through the enlargement of the electron depletion layer. This amplification of the gas response was further aided by the formation of a p–n heterojunction between the GQDs and the SnO_2_ surface, which improved charge transport and electrical properties. In comparison to the graphene-based NO_2_ gas sensor discussed earlier, this study demonstrated that GQDs with discrete band gaps can effectively enlarge the electron depletion layer on the surface. This enlargement leads to highly sensitive NO_2_ sensing with an ultralow detection limit of 1.1 ppb and quick recovery times (105 s).

### 5.2. Detection of HCHO Gas

Chen et al. studied the HCHO gas sensing performance of pure ZnO and a graphene-doped ZnO composite (G–ZnO–2) synthesized by an in situ method [122]. Compared to the bare ZnO, the G–ZnO–2 exhibited excellent HCHO sensing properties such as a higher response to 100 ppm HCHO gas (R_a_/R_g_ = 12), faster response/recovery time (10 s/29 s), and good selectivity at an optimal working temperature of 200 °C. In the G–ZnO–2 composite, the graphene acted as an electron acceptor to increase the depletion layer of ZnO. Therefore, compared with the pure ZnO, the composite showed a larger change in resistance and reduced the response time. In addition, the high electrical conductivity of the graphene and its strong electronic interactions with ZnO promoted the effective transfer of electrons, thereby enhancing the sensing performance. Other research has investigated the utilization of mesoporous ultrathin SnO_2_ modified with N–GQDs (N–GQDs/SnO_2_) for HCHO detection [63]. With the addition of 1.00 wt% N–GQDs, the response (R_a_/R_g_) of the SnO_2_ gas sensor increased from 120 to 361 at 60 °C for the detection of 10 ppm HCHO. Different functional groups on N–GQDs, including carboxyl and amino groups, provided more adsorption sites for gas molecules and tuned the electrical conductivity and electron transport properties of the material. When the N–GQDs and SnO_2_ nanosheets contacted each other, electrons were transferred from the low work function (N-GQDs = 5.22 eV) to the high work function (SnO_2_ = 5.32 eV), and a Schottky barrier was formed between the N–GQDs and SnO_2_. Consequently, the electron concentration in the N–GQDs/SnO_2_ increased, enhancing its gas-sensing performance. Compared to the previously discussed G–ZnO–2 nanocomposite, the N–GQDs/SnO_2_ demonstrated several noticeable results, including a higher response to 10 ppm HCHO with a sensitivity that was three times higher than that of the pristine metal oxide at a lower operating temperature (60 °C) and a lower detection limit of 0.01 ppm. The enhanced sensing properties of the N–GQDs were attributed to the abundant functional groups on its surface, larger adsorption sites, and efficient electronic regulation to SnO_2_ nanosheets.

**Figure 11:**
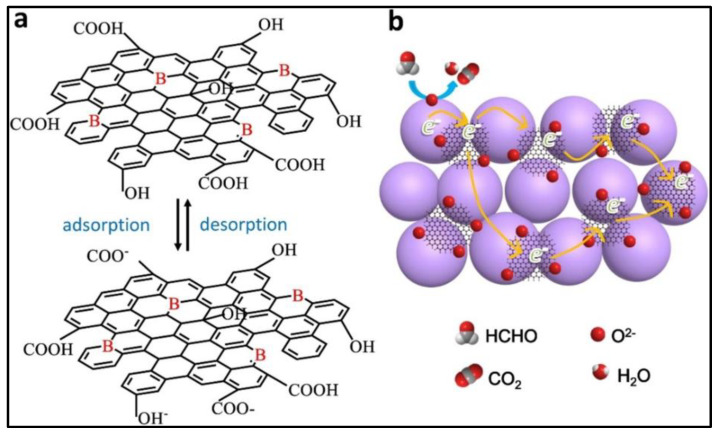
Schematic sensing mechanism of (**a**) B–GQD and (**b**) B–GQDs–AL [95].

Zhang et al. combined AL with B–GQD to enhance the HCHO gas-sensing properties [95]. The operating temperature decreased from 90 °C to 55 °C with the incorporation of the GQDs into AL. Moreover, rapid response times (20–35 s) and recovery times (30–130 s) were achieved as the HCHO concentration increased from 1 to 30 ppm. The -COOH functional groups on the surface of the B–GQD ionized into COO- and H+, which then reacted with HCHO gas to form electrons, thus increasing the gas response (Rg/Ra) as shown in Figure 11a. When the B–GQD was hybridized with AL, the B–GQD formed a bridge between the AL grain boundaries, facilitating electron transfer when exposed to HCHO gas molecules and resulting in an enhanced response (Figure 11b).

### 5.3. Detection of NH_3_ Gas

**Figure 12:**
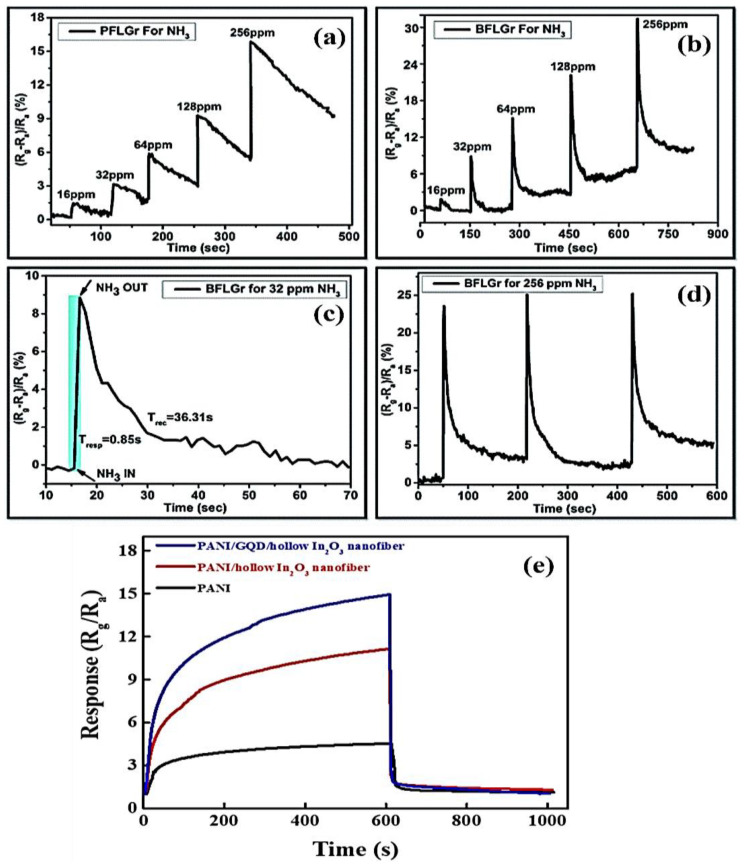
Response vs. time plots for (**a**) PFLGr and (**b**) BFLGr for 16 to 256 ppm of NH_3_; (**c**) response and recovery plot for the BFLGr sensor for 32 ppm of NH_3_; (**d**) repeatability plot for BFLGr for 256 ppm of NH_3_ [123]; (**e**) the response curves of the PANI polymer matrix, 20 wt% PANI/hollow In_2_O_3_ nanofiber, and 20 wt% PANI/GQD/hollow In_2_O_3_ nanofiber composites with an exposure of 1 ppm NH_3_ at room temperature [124].

Srivastava et al. explored the NH_3_ gas-sensing properties of pure few-layer graphene (PFLGr) and boron-doped few-layer graphene (BFLGr) nanosheets through a low-pressure chemical vapor deposition method [123]. The response values for the BFLGr and PFLGr sensors were 8.92% and 2.64%, respectively, for 32 ppm of gas as shown in Figure 12a,b. This result implies that boron doping in graphene improves the interaction of the nanosheets with the NH_3_ gas molecules. The response time for the BFLGr sensor was 0.85 s, which is much less as compared to the undoped PFLGr sensor (3.56 s) shown in Figure 12c, while the recovery times for the sensors were 36.31 s and 48.24 s, respectively. It can be clearly seen from Figure 12d that the BFLGr sensor exhibits high repeatability over three response–recovery cycles. The higher adsorption energy for NH_3_ on BFLGr (−0.50 eV) is attributed to the strong interaction between the electron-deficient boron atom and the electron-offering N atom of the NH_3_ molecule as compared to that of PFLGr (−0.24 eV).

Indium et al. fabricated a new ternary nanocomposite based on the conducting polymer PANI, hollow In_2_O_3_ nanofiber, and N–GQD as a NH_3_ gas sensor using in situ chemical oxidative polymerization [124]. The response of the PANI/N–GQD/hollow In_2_O_3_ nanofiber sensor, with a 20 wt% loading of N–GQD-coated hollow In_2_O_3_ nanofiber, reached 15.2 when exposed to 1 ppm NH_3_, marking an increase of over 4.4 times as compared to the PANI sensor (Figure 12e). This ternary composite sensor has demonstrated exceptional sensitivity in NH_3_ detection within a concentration range of 0.6 ppm to 2.0 ppm at room temperature, a crucial capability for the early detection of hepatic or kidney diseases through human breath analysis. The highly sensitive detection of low concentrations of NH_3_ can be attributed to the p–n heterojunctions formed between the p-type PANI and n-type N–GQD-coated hollow In_2_O_3_ nanofibers. Notably, the PANI/N–GQD/hollow In_2_O_3_ nanofiber sensor outperforms the previously described PFLGr and BFLGr sensors in NH_3_ sensing at room temperature. This superior performance can be attributed to the presence of oxygen-containing defects and the extensive special surface area of N–GQDs, which enhance the contact sites with PANI and provide a considerable number of adsorption sites for NH_3_ gas.

### 5.4. Detection of H_2_S Gas

Shao et al. developed a GQD-decorated hierarchical SnO_2_ quantum NPs (SnO_2_QNP)/ZnO nanostructures via a self-assemble strategy for the detection of H_2_S gas [115]. In comparison to bare ZnO and SnO_2_/ZnO sensors, the GQD–SnO_2_QNP/ZnO sensor demonstrated a significantly elevated response (S = 15.9 for 0.1 ppm H_2_S) and a rapid response/recovery time (14/13 s), along with notable selectivity toward H_2_S over other interfering gases. This enhancement is primarily contributed to the strong synergistic effect and p–n heterojunction between the p-type GQD and the n-type SnO_2_ and ZnO, effectively amplifying the resistance variation due to the change in oxygen adsorption. The combined effects of GQD/SnO_2_QNP/ZnO heterointerfaces contributed to the improvement of the selectivity of the sensors, indicating considerable potential for non-invasive exhaled diagnosis. Hsu et al. prepared CuO-doped ZnO nanofibers (CuO/ZnO NFs) using a sol-gel method and an electrospinning method for H_2_S gas-sensing studies [113]. At 200 °C, the CuO/ZnO NFs exhibited a higher gas response (83.98%) when exposed to 1 ppm H_2_S compared to that of the pristine ZnO NFs (25.79%), with good recovery and reproducibility. During the oxidation–reduction process, when the CuO-doped ZnO gas sensing materials are exposed to the air, the oxygen molecules can easily adsorb onto the surface and capture free electrons to form O_2_^−^ species. The O_2_^−^ species can then effectively react with the contacted H_2_S gas molecules, thereby facilitating the gas-sensing performance of the CuO/ZnO NFs.

### 5.5. Detection of Ethanol Gas

Rahimi et al. Drop-casted GQDs synthesized through the pyrolysis of citric acid, onto a ZnO nanorod (ZnO NR) thin film to enhance the sensitivity of ZnO NR toward ethanol gas [93]. It was observed that the sensitivity of the GQD–ZnO NR thin film (approximately 75%) is significantly higher than that of the bare ZnO NR thin film (around 10%) when exposed to 500 ppm ethanol gas. The authors proposed two major roles of the GQDs contributing to this enhancement: (1) GQDs could promote the adsorption of oxygen and ethanol gas molecules, and (2) GQDs could provide a path for transfering electrons by providing interconnections among the ZnO nanorods. Due to their high electron mobility, GQDs can facilitate the transport of charge carriers, thus improving their gas sensitivity. Lei et al. incorporated Au with ZnO to optimize and enhance the electron transfer efficiency of the mesoporous ZnO nanospheres during sensing of ethanol gas [108]. The prepared Au–ZnO nanosphere demonstrates its highest response to ethanol (approximately 159 for 50 ppm) at 200 °C. The enhanced sensor performance is attributed to the open mesopores and the textured surface of the hybrids which facilitate the adsorption and desorption of ethanol molecules on the sensing layers. The addition of Au nanoparticles produced more activated oxygen species on the surface of the mesoporous ZnO, which could adsorb more ethanol molecules to participate in the catalytic reaction. Therefore, the strong spillover effect of the Au nanoparticles increases the electron transfer rate and promotes surface catalytic oxidation, contributing to the improved performance of the sensor. Moreover, the authors compared the gas-sensing performance of the ZnO–Au that was synthesized using different methods. The optimal responses obtained for the mesoporous ZnO−Au prepared by NaBH_4_ reduction, photoreduction, and H_2_ reduction are 107, 159, and 65, respectively. This suggests that the size of Au nanoparticles can be easily tuned by changing reduction strategies, and that the size effect plays a crucial role in gas sensing, thereby influencing various catalytic activity and grain aggregation.

### 5.6. Detection of Acetone Gas

Chu et al. prepared SnO_2_/GQDs nanocomposites via the solvothermal method to detect acetone vapor [91]. They observed that a strong response to 1000 and 0.1 ppm acetone reached 120.6 and 1.3, respectively, whereas the response of pure SnO_2_ to 1000 ppm acetone was only 2.3. The authors proposed that the improved response could be attributed to the establishment of a heterojunction between the SnO_2_ and GQDs. The incorporation of GQDs into the SnO_2_ matrix notably enhanced the electronic conduction within the composite. Additionally, the defects and oxygen-containing groups present on the surface of GQDs served as effective adsorption sites for acetone gas molecules, thereby amplifying the responses of the SnO_2_/GQDs composites. Lu et al. employed a facile ZnSn(OH)_6_-sacrificial template method to fabricate mesoporous hollow Zn_2_SnO_4_/SnO_2_ microboxes for acetone gas sensing [120]. Compared with the pure SnO_2_ sensor, the Zn_2_SnO_4_/SnO_2_ sensor displayed not only a two times higher response (20.1) toward 100 ppm acetone but also excellent selectivity and stability at the optimal operating temperature of 250 °C. The enhanced sensing performance can be mainly attributed to the heterojunction formed between the SnO_2_ and Zn_2_SnO_4_ and the unique “mesoporous hollow structure”. Due to the higher conduction band edge potential of the Zn_2_SnO_4_ as compared to that of SnO_2_, electrons from the Zn_2_SnO_4_ migrate to the conduction band of the SnO_2_ until the system’s Fermi level reaches equilibrium. This process results in the separation of charges at the interface, providing more electrons to oxygen than pure SnO_2_ would. Consequently, a more noticeable change in resistance occurs, leading to an enhanced gas-sensing response. Moreover, the distinctive “mesoporous hollow structure” offers significant advantages for promoting acetone gas diffusion within the Zn_2_SnO_4_/SnO_2_ sensor, leading to more efficient absorption of acetone molecules on both sides of the porous shell of Zn_2_SnO_4_/SnO_2_ microboxes. This enhances their reactivity at the surface level, consequently improving gas sensitivity.

## 6. Current Challenges of GQD-Based Gas Sensors

Over the past few years, researchers have achieved some advancements; however, GQD-based gas sensors still face significant challenges in terms of industrial manufacturing and the mitigation of air pollutants. Although GQDs show promise in terms of sensitivity, improving their sensitivity to trace levels of target gases is an ongoing challenge, particularly for applications requiring ultralow detection limits. Furthermore, gas-sensing stability is an important index for practical gas sensors. Addressing the influence of environmental factors, such as humidity, temperature, and interference from other gases on the sensor’s performance is critical for the reliable operation and long-term stability of GQD-based sensors. In particular, high humidity can lead to the absorption of water molecules on the surface of the sensor, potentially altering its electrical properties and interfering with the gas-detection process. Prolonged exposure to environmental factors, such as humidity and temperature fluctuations, can affect the long-term stability of GQD-based sensors, which may lead to sensor drift or degradation. The development of encapsulation techniques, protective coatings, and robust housing for GQD-based sensors can help to maintain their performance and extend their operational lifespan, especially in harsh environments. Finally, bridging the gap between research and commercialization, as well as establishing standardization protocols for GQD-based gas sensors, are essential for these sensors’ widespread adoption in industrial and consumer markets. Addressing these challenges is fundamental for unlocking the full potential of GQD-based gas sensors for various applications, including environmental monitoring, industrial safety, and healthcare.

In addition, NPs also exhibit limitations in gas-sensing applications. One significant limitation is their susceptibility to agglomeration because NPs tend to cluster together, affecting their dispersibility and, consequently, the uniformity of the sensing material. This aggregation can lead to decreased surface area and hinder effective interaction with gas molecules, impacting the sensor’s overall performance. Additionally, issues related to stability, reproducibility, and the potential toxicity of certain nanoparticles can pose challenges in long-term and widespread use for gas-sensing purposes. Addressing these limitations is crucial for advancing the effectiveness and reliability of nanoparticle-based gas sensors.

## 7. Conclusions and Future Perspectives

Increasing urban population, industrial emissions, and vehicle exhaust emissions are the primary sources of air pollutants that regularly harm the natural environment. It is essential to monitor these air pollutants continuously to prevent damage to human health and environmental deterioration. Effective, existing monitoring instruments, however, tend to be time-consuming, expensive, and seldom-employed for real-time monitoring. In recent years, significant research has been conducted within the scientific community to develop an ideal environmental sensor, bridging the gap between theoretical concepts and practical implementation. In this review, we have focused on the latest developments of GQD-based gas sensors, along with the construction methods of sensing materials. The sensing mechanisms of GQD-based gas sensors are presented, and the roles of GQDs in enhancing gas-sensing performance are comprehensively discussed. The quantum confinement behavior and electron modification of GQDs have become significantly appealing, particularly when compared to graphene. Although research on GQD-based gas sensors has made significant progress in recent years, additional efforts are needed to improve the key detection parameters, such as sensitivity and selectivity.

To enhance the performance of GQD-based gas sensors, it is crucial to determine their optimal sizes and shapes. For instance, increasing the surface-to-volume ratio by tailoring the morphology of the sensing materials can effectively boost the specific surface area. The incorporation of GQDs with noble metals, other oxides, polymers, and metal-organic frameworks can enhance selectivity, sensitivity, thermal stability, and response and recovery times. Consequently, the proper selection of dopants and additives can effectively reduce cross-sensitivity to environmental factors, such as humidity, and ultimately improve the overall sensing performance. Furthermore, it is essential to emphasize the significance of simulation-based theoretical investigations, such as density functional theory, in order to advance our understanding of the sensing mechanisms. This deeper understanding will facilitate the development of GQD-based gas-sensor compositions that offer enhanced precision for tuning their properties, resulting in substantial improvements. Moreover, researchers should extensively explore the potential of GQD-based sensors for human volatomics-based noninvasive, painless, and point-of-care disease diagnosis and health monitoring. Surprisingly, research on GQD-based gas sensors for human health monitoring is scarce, making it a subject worthy of further investigation. In addition, incorporating neural networks and artificial intelligence (AI) into GQD-based gas sensors is an exciting prospect in the field of gas sensing. The incorporation of neural networks and AI algorithms has the potential to revolutionize the way GQD-based gas sensors function. These advanced technologies offer real-time data analysis, pattern recognition, and adaptive response mechanisms, ultimately leading to improved accuracy, sensitivity, and selectivity. We hope that this review provides guidance for future research on functionalized GQDs and GQD-based nanocomposites for gas sensing applications.

## Figures and Tables

**Table 1:** A summary of selected GQD-based gas sensors with different construction strategies.

	GQDs Based Sensor	Preparation Method	Target Gas	Ref.
Elemental doping	N-GQDs	Hydrothermal	Formaldehyde	[59]
Edge functionalization	OH-GQDs	Hydrothermal	Ammonia	[18]
Composite	ZnCo_2_O_4_/GQD	Hydrothermal	Triethylamine	[60]
	GQD@SnO_2_	Drop casting/coating	NO_2_	[61]
	MoS_2_/rGO/GQDs	Hydrothermal	NO_2_	[62]
	CoPc–GQD	π–π stacking	DMMP	[63]
	ZnO:GQDs	Drop casting/coating	Ammonia	[64]
Elemental doping and composite	NGQDs/PANI	Chemical oxidative polymerization	Ethanol	[65]
	N-GQDs@SnO_2_	Vigorous stirring	NO_2_	[66]
	N-GQDs@ZnO	Hydrothermal	NO_2_	[67]
	N-GQDs@SnO_2_	Ultrasonic impregnation	Formaldehyde	[68]

ZnCo_2_O_4_: zinc cobaltite; SnO_2_: tin oxide; rGO: reduced graphene oxide; MoS_2_: molybdenum disulfide; PANI: polyaniline; DMMP: dimethyl methylphosphonate.

**Table 2:** The roles of GQDs in improving the sensing performance of GQD-based gas sensors.

Role of GQDs	GQDs Based Sensor	Target Gas	Conc. (ppm)	Operating Temp.	Sensitivity/Response	T_res_/T_rec_	Ref.
Strong interaction with analyte	ZnO:GQDs	NH_3_	1000	RT	6047	170/80 s	[64]
	N-GQDs@SnO_2_	HCHO	100	60 °C	256	<12/12 min	[34]
	OH-GQDs	NH_3_	500	RT	76.63%	64/69 s	[18]
	TiO_2_/af-GQDs	H_2_S	55	RT	26.62	68/77 s	[88]
Formation of heterojunction	TiO_2_@NGQDs	NO	100	RT	~31.1%	235/285 s	[86]
	B/APPH	Benzene	1	65 °C	17.5	-	[87]
Increased surface area	ZnCo_2_O_4_/GQD	TEA	100	200 °C	6.97	45/65	[60]
	NGQDs/PANI	Ethanol	100	RT	0.66%	85/62 s	[65]
Protecting layer	MoS_2_/rGO/GQDs	NO_2_	5	RT	15.2%	150/150 s	[62]
Protecting layer and heterojunction formation	GQDs/SiNW	NO_2_	10	RT	~17	-	[89]
Increased surface area and heterojunction formation	ZnFe_2_O_4_-GQDs	Acetone	5	RT	1.2	<12/12 s	[90]
	ZnO/S, N: GQDs/PANI	Acetone	0.5	RT	2	15/27 s	[72]
Strong interaction with analyte and heterojunction formation	GQD@SnO_2_	NO_2_	5	RT	4.8	322 s/105 s	[61]
	N-GQDs/SnO_2_	HCHO	10	60 °C	361	330 and 30 s	[68]
	N-GQDs@ZnO	NO_2_	5	100 °C	57	180/100 s	[67]
	N-GQDs@SnO_2_	NO_2_	1	130 °C	417	59/33 s	[66]
	N-GQDs/3DOM In_2_O_3_	NO_2_	1	100 °C	81.7	59/43 s	[73]
	N-GQDs@SnO_2_	NO_2_	0.01	150 °C	292	181/81 s	[69]
	SnO_2_/GQDs	Acetone	1000	RT	120.6	17/13 s	[91]
-	CoPc–GQD	NO_2_	50	RT	15.8	1.67/1.67 min	[92]
-	GQD-ZnO	Ethanol	500	RT	~75	-	[93]
-	GQDs-ZnO	Acetic acid	1	RT	~15	11/12 s	[94]
-	B-GQD/AL	HCHO	1	65 °C	18	23/30 s	[95]
-	CoPc–HFIP–GQD	DMMP	20	RT	8.4%	600/640 s	[63]
-	CoPc–6FBPA–GQD	DMMP	20	RT	9.3%	600/620 s	[63]

Conc.: concentration; Operating Temp.: operating temperature; T_res_: response time_;_ T_rec_: recovery time; RT: room temperature; TEA: triethylamine; HCHO: formaldehyde; DMMP: dimethyl methylphosphonate; 3DOM: three-dimensional ordered microporous; NH_3_: ammonia.

**Table 3:** The gas-sensing performances of graphene, GQDs, and other NP-based sensors.

Gas Sensor	Target Gas	Conc. (ppm)	Operating Temp.	Sensitivity/Response(Ra/Rg)	T_res_/T_rec_	Ref.
Ce/SnO_2_	NO_2_	1	140 °C	42.15	-	[101]
Pt-MoSe_2_		20	RT	7.79	32 s/-	[102]
GaN QDs		100	RT	52.23%	47/119 s	[103]
ZnO@CNF	NH_3_	50	RT	12.3%	5/18 s	[104]
CuO/ZnO		1	RT	1.59	2.3/2.1 s	[105]
SnO_2_/WSe_2_		5	RT	87.07%	24/40 s	[106]
CeO_2_/ZnSnO_3_	Ethanol	100	200 °C	219.2	12/22 s	[107]
ZnO−Au		50	200 °C	159	-	[108]
GR/In-ZnO	HCHO	10	RT	1891%	-	[109]
Co-SnO_2_		30	90 °C	163.44	652/475 s	[110]
NiS/Ni-ZnO		10	RT	330%	39.4/40.7 s	[111]
Au/SnO–SnO_2_	H_2_S	100	240 °C	85.27%	22/63 s	[112]
CuO-ZnO		1	200 °C	83.98%	9/160 s	[113]
Zn-SnO_2_		1	70 °C	0.35	96/123 s	[114]
GQD-SnO_2_QNP/ZnO		0.1	RT	15.9%	14/13 s	[115]
Au@Co_3_O_4_	Acetone	10	250 °C	27.05%	233/280 s	[116]
Fe–ZnO		100	365 °C	105.7	-	[117]
In_2_O_3_/ZrO_2_		100	260 °C	60.38	1/41 s	[118]
Ru-doped SnO_2_		100	250 °C	340	0.58/8.4 s	[119]
Zn_2_SnO_4_/SnO_2_		100	250 °C	20.16	97/315 s	[120]

## Data Availability

Not applicable.
